# Biotransformation of Lignin by an Artificial Heme Enzyme Designed in Myoglobin With a Covalently Linked Heme Group

**DOI:** 10.3389/fbioe.2021.664388

**Published:** 2021-05-31

**Authors:** Wen-Jie Guo, Jia-Kun Xu, Jing-Jing Liu, Jia-Jia Lang, Shu-Qin Gao, Ge-Bo Wen, Ying-Wu Lin

**Affiliations:** ^1^School of Chemistry and Chemical Engineering, University of South China, Hengyang, China; ^2^Key Lab of Sustainable Development of Polar Fisheries, Ministry of Agriculture and Rural Affairs, Yellow Sea Fisheries Research Institute, Chinese Academy of Fishery Sciences, Lab for Marine Drugs and Byproducts of Pilot National Lab for Marine Science and Technology, Qingdao, China; ^3^Laboratory of Protein Structure and Function, University of South China Medical School, Hengyang, China

**Keywords:** protein design, heme protein, artificial enzyme, lignin, degradation

## Abstract

The conversion of Kraft lignin in plant biomass into renewable chemicals, aiming at harvesting aromatic compounds, is a challenge process in biorefinery. Comparing to the traditional chemical methods, enzymatic catalysis provides a gentle way for the degradation of lignin. Alternative to natural enzymes, artificial enzymes have been received much attention for potential applications. We herein achieved the biodegradation of Kraft lignin using an artificial peroxidase rationally designed in myoglobin (Mb), F43Y/T67R Mb, with a covalently linked heme cofactor. The artificial enzyme of F43Y/T67R Mb has improved catalytic efficiencies at mild acidic pH for phenolic and aromatic amine substrates, including Kraft lignin and the model lignin dimer guaiacylglycerol-β-guaiacyl ether (GGE). We proposed a possible catalytic mechanism for the biotransformation of lignin catalyzed by the enzyme, based on the results of kinetic UV-Vis studies and UPLC-ESI-MS analysis, as well as molecular modeling studies. With the advantages of F43Y/T67R Mb, such as the high-yield by overexpression in *E. coli* cells and the enhanced protein stability, this study suggests that the artificial enzyme has potential applications in the biodegradation of lignin to provide sustainable bioresource.

## Introduction

**FIGURE 1:**
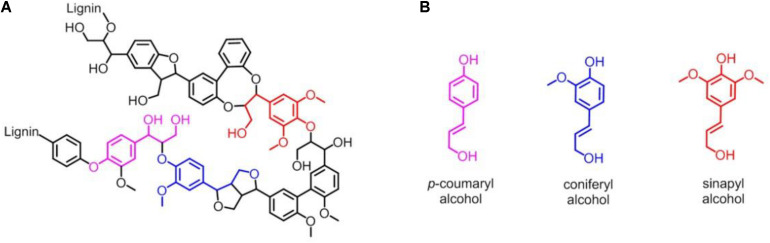
**(A)** Representative chemical structure of lignin. **(B)** Three major monomers of lignin: *p*-coumaryl alcohol, coniferyl alcohol and sinapyl alcohol unit, respectively.

Lignin accounts for 10∼35% by weight, up to 40% by energy in biomass, and lignin is also by far the most abundant renewable source composed of aromatic units in nature (Leonowicz et al., 1999; Paliwal et al., 2012). As shown in Figure 1A, lignin is chemically a cross-linked phenolic polymer (Yoshikawa et al., 2013), and its chemical structure contains three major monomers, *p*-coumaryl alcohol, coniferyl alcohol, and sinapyl alcohol units (Figure 1B), which are linked by carbon-carbon and carbon-oxygen bonds (Picart et al., 2015). The conversion from lignin to chemicals and/or fuels can be traced back to the 1930s, and various lignin depolymerization methods have being explored from academia to industry worldwide (Zakzeski et al., 2010a; Li et al., 2015; Upton and Kasko, 2016; Chan et al., 2020; Zhang and Wang, 2020). However, although the industrial production of lignin is 5–36 billion tons per year, it is still challenging to efficiently convert into aromatics, and only ∼2% is used in industry, such as for generating binder, surfactant, chelating agent and paper pulp, with the rest burned to produce energy (Zakzeski et al., 2010b; Zhang et al., 2011; Chan et al., 2020; Zhang and Wang, 2020). Therefore, it is urgent to develop new technology for utilizing the lignin with high efficiency in industry (Kamimura et al., 2019).

To date, various chemical methods have been developed for degradation and transformation of lignin (Zakzeski et al., 2010a; Li et al., 2015; Upton and Kasko, 2016; Zhang and Wang, 2020), such as by the pyrolysis of lignin at high temperature (Davaritouchaee et al., 2020), and by the reaction with ozone (Stergiou et al., 2008). Meanwhile, the harsh conditions such as strong acids/bases (H_2_SO_4_/NaOH) and high temperature (125–320°C)/pressure (0.5–2 MPa) make it difficult to treat the biomass in downstream processes (Banerjee et al., 2010). Comparing to the traditional chemical methods, microbial degradation and enzymatic catalysis provide a gentle way for the degradation of lignin (Bugg et al., 2020; Chan et al., 2020; Chauhan, 2020). The common enzymes (biological catalysts in living organisms) capable of lignin degradation are laccases and peroxidases (lignin peroxidase, manganese peroxidase, and dye-decolorizing peroxidase, etc.), which are copper-containing and heme-containing enzymes, by using O_2_ and H_2_O_2_ as an oxidant, respectively (Singh et al., 2013; Chen et al., 2015; Lin, 2020, 2021). Note that peroxidases utilize H_2_O_2_ or other peroxides in the one-election oxidation of various cosubstrates, whereas peroxygenases insert one of the O atom from the oxidant to the substrates, by using the same catalytic intermediate, Compound I (an oxoferryl heme π-cation radical) (Hrycay and Bandiera, 2012; Wang et al., 2017).

Cytochromes P450 (CYP450) are a large class of heme-containing monooxygenases catalyzing the incorporation of one atom from O_2_ into organic substrates (Denisov et al., 2005). They also exhibit the ability of lignin degradation, however, require the expensive cofactor (such as nicotinamide adenine dinucleotide phosphate, NADPH) (Mallinson et al., 2018). In addition to the pathway of O_2_ activation, CYP450 may use the H_2_O_2_ shunt, as that of peroxidase/peroxygenase, to generate the catalytic intermediate, Compound I (Denisov et al., 2005). By protein engineering, a series of CYP450 variants were constructed to transform lignin into small aromatic compounds using H_2_O_2_ as the oxidant, with the help of decoy molecules (Mallinson et al., 2018; Xu et al., 2019; Ariyasu et al., 2020; Jiang et al., 2020).

**FIGURE 2:**
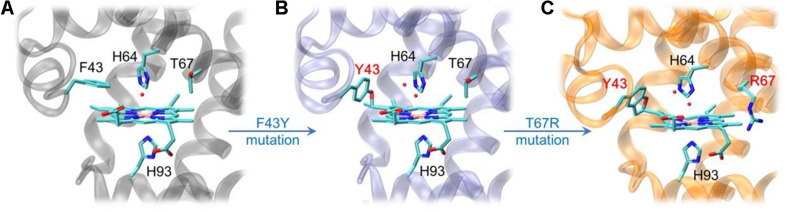
X-ray crystal structures of wild-type (WT) Mb [**A**, PDB code 1JP6 (Urayama et al., 2002)], F43Y Mb [**B**, PDB code 4QAU (Yan et al., 2015)], and F43Y/T67R Mb [**C**, PDB code 6JP1 (Liu et al., 2019)], respectively, showing the heme active site and Tyr-heme cross-links.

By rational modification of the heme center of myoglobin (Figure 2A), an O_2_ carrier, we obtained several artificial heme enzymes, including the artificial dehaloperoxidases, dye-decolorizing peroxidases and DNA nucleases, etc. (Yin et al., 2018; Zhang et al., 2019, 2020; Luo et al., 2020). By introducing a Tyr residue (F43Y mutation) in the heme distal pocket, we discovered a new post-translational modification (PTM) of heme protein, i.e., a Tyr-heme cross-link, in the F43Y Mb mutant (Figure 2B; Yan et al., 2015), which is distinct from those observed for other heme enzymes (Lin, 2015, 2018). To mimic the heme active of both natural peroxidase and CYP450 those contain a conserve His-Arg pair acting as an acid-base catalyst (Poulos, 2014), we constructed a double mutant of F43Y/T67R Mb by further introducing a distal Arg67 (Liu et al., 2019). Interestingly, we discovered novel Tyr-heme double cross-links in the double mutant (Figure 2C), which improve the protein stability. Moreover, the double mutant exhibited considerably enhanced peroxidase activity, by catalyzing the oxidation of various phenolic molecules, which is comparable to those of the natural peroxidases (Liu et al., 2019; Liao et al., 2020). Therefore, we envisaged that the double mutant might also have the ability to depolymerize lignin.

To test our speculation, we herein investigated the depolymerization of the model lignin dimer GGE and Kraft lignin by the artificial enzyme of F43Y/T67R Mb. The tolerances of organic solvents and H_2_O_2_ for the enzyme were studied. Kinetic UV-Vis studies were performed, and the reaction products were identified by UPLC-ESI-MS analysis. Moreover, a molecular modeling study was performed for GGE binding to the enzyme, and a possible catalytic mechanism was also proposed and discussed.

## Materials and Methods

### Materials

The peroxidase substrates, 2,2-Azino-bis (3-ethylbenzothiazoline-6-sulfonic acid) (ABTS) and guaiacol were bought from Aladdin Industrial Corporation (Shanghai, China). Guaiacylglycerol-β-guaiacyl ether (GGE) was purchased from Shanghai Macklin Biochemical Co., Ltd (Shanghai, China). Kraft lignin was bought from Sigma-Aldrich (United States). Wild-type (WT) sperm whale Mb and the double mutant of F43Y/T67R Mb were expressed and purified as previously reported (Liu et al., 2019).

### Peroxidase Activity and Kinetic Measurements

To investigate the enzyme stability, the effects of organic solvent species or concentrations on the peroxidase activity of F43Y/T67R Mb were investigated on a stopped-flow spectrophotometer (SF-61DX2 Hi-Tech KinetAsyst^TM^ ) at room temperature, by using ABTS as the substrate and H_2_O_2_ as the oxidant, respectively. We used ethanol or dimethyl sulfoxide (DMSO) as the organic solvent, and tested the solubility of GGE or Kraft lignin by addition of 5–50% (v/v). The effect of different concentrations of organic solvent on activity of F43Y/T67R Mb with 0.1 mM ABTS was determined by using the standard UV-Vis kinetic assay at 25°C, as reported previously (Liu et al., 2019).

Typically, one syringe contains 4 μM protein (in 100 mM potassium phosphate buffer, pH 5.5) in presence of 0.2 mM ABTS, and different amount of ethanol or DMSO (5–20%, v/v), and the second syringe contains 4 mM H_2_O_2_, as determined with ε_240__ nm_ = 39.4 M^–1^⋅cm^–1^. Upon mixing, the reaction was monitored by formation of the ABTS^+.^ cation radical at 660 nm. The initial rate was calculated based on the initial linear changes using an extinction coefficient of ε_660__*nm*_ = 14.0 mM^–1^.cm^–1^ (Nastri et al., 2011). The H_2_O_2_-dependent of the peroxidase activities were performed under the similar conditions by varying the concentrations of H_2_O_2_ (0∼100 mM). The plot of initial rates versus the concentrations of H_2_O_2_ was fitted to the Hill equation: Rate/[protein (heme unit, μM)] = *k*_*cat*_[H_2_O_2_]^*h*^/[(*K*_1__/__2)_^*h*^ + [H_2_O_2_]^*h*^] (Zhang et al., 2019).

### Oxidation of GGE Catalyzed by F43Y/T67R Mb

#### UV-Vis Spectroscopy

UV-Vis spectra were recorded on a Hewlett-Packard 8453 diode array spectrometer. Titration of F43Y/T67R Mb with GGE was performed at 25°C. The UV-Vis spectra were recorded in a range of 200–700 nm with drop wise addition of GGE to a final concentration of ∼0.1 mM in the absence or presence of H_2_O_2_.

#### Product Analysis by UPLC-ESI-MS

The enzymatic oxidation products of GGE were monitored by Ultra Performance Liquid Chromatography (UPLC) ESI-MS. At first, the reaction mixtures (2 mL) contained 2.5 mM of GGE dissolved in 50% ethanol (v/v), 2.0 mM H_2_O_2_, and 5.0 μM F43Y/T67R Mb in potassium phosphate buffer (pH 5.5). After reaction for 1 h, aliquots were withdrawn and diluted in acetonitrile (1:1 ratio), and then analyzed in a Waters ACQUITY UPLC/Xevo G2 QTOF system, using a reverse-phase C-18 column (ACQUITY UPLC^®^BEH C18 1.7 μm, 2.1 mm × 50 mm) with a precolumn at 40°C and a flow rate of 0.5 mL/min (Liu et al., 2018). The column was further equilibrated with the mobile phase of 70% water (eluent A)/30% acetonitrile (eluent B) for 5 min. The mass spectrometer was operated in the ESI positive ion modes.

### Molecular Docking Study

The X-ray structure of ferric F43Y/T67R Mb, as reported in our previous study (Liu et al., 2019), was used as the initial structure for docking with GGE using the Autodock 4.0 (Morris et al., 2009). The heme iron was set to be the center, with a box size of ∼60 Å × 60 Å × 60 Å, which covered most of the protein surface. Amino acid residues Arg45, Asp60, Lys63, Arg67, and Lys96 on the protein surface near the active heme site were set as flexible residues. GGE as a substrate for docking were generated using the Dundee Prodrg2 server (Schuttelkopf and van Aalten, 2004). Docked conformations were ranked automatically by Autodock 4.0 using a binding-energy scoring function. The docking results (10 most favorable conformations) after 2,000 steps were then visualized and analyzed using VMD 1.9 (Humphrey et al., 1996).

### Oxidation of Kraft Lignin Catalyzed by F43Y/T67R Mb

#### Kinetic UV-Vis Study

In a 2 mL cuvette, Kraft lignin (10 mg sample was dissolved in 1 mL DMSO) (2–20 μM), potassium phosphate buffer (1,400 μL, pH 5.5, 100 mM), DMSO (5%, v/v), F43Y/T67R Mb or WT Mb (400 μL, 2 μM), and H_2_O_2_ (100 μL, 2 mM) were mixed in the order stated, and the absorbance was monitored at 465 nm for 5–10 min (Rahmanpour et al., 2016). The molar concentration of Kraft lignin was calculated using an average molecular mass of 10,000 Da. The observed rate constants (*k*_*obs*_) were calculated by fitting the absorbance at 465 nm versus time to a single-exponential decay function (Chen et al., 2021).

#### ESI-MS Assay of Low Molecular Weight Products

Mass spectra of the oxidation products of Kraft lignin were carried out on G2-XS QTOF mass spectrometry (Waters). Kraft lignin (10 mg) was dissolved in DMSO (4 mL), and 200 μL of the sample was added to potassium phosphate buffer (2 mL, 100 mM, pH 5.5), followed by addition of F43Y/T67R Mb (2 μM) and H_2_O_2_ (2 mM). The resulting solution was incubated at room temperature for 1 h. The reaction was stopped by adding 1 M HCl (10 μL), and reaction products were extracted into two volumes of ethyl acetate, and then the solution was centrifuged for 5 min at 11, 962 *g*. Supernatant was removed, evaporated and the precipitate was dissolved in 1.5 mL acetonitrile. Then reaction samples were transferred into the mass spectrometer chamber for measurement under a positive mode.

## Results and Discussion

### Effects of Solvent/H_2_O_2_ on the Enzymatic Activity

**FIGURE 3:**
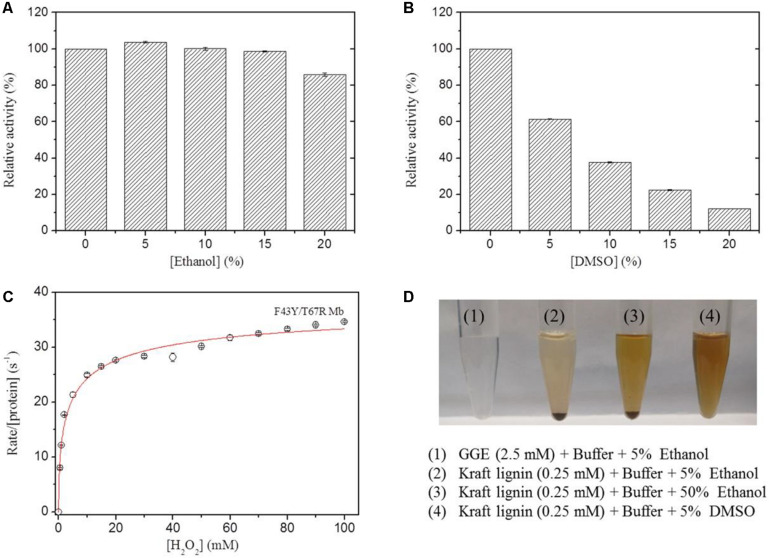
Effects of ethanol **(A)** and DMSO **(B)** concentrations on the peroxidase activity of F43Y/T67R Mb, determined by measuring the oxidation of ABTS at pH 5.5 and room temperature. The activity in the absence of the organic solvent was taken as 100%. Values represent the means of three independent experiments (mean ± standard error). **(C)** Steady-state rates of H_2_O_2_-dependent oxidation of ABTS catalyzed by F43Y/T67R Mb, as a function of H_2_O_2_ concentrations. The plot was fitted to the Hill equation. Reaction conditions: 2 μM protein, 0.1 mM ABTS and 50 mM potassium phosphate buffer at pH 5.5. **(D)** Visual appearance of GGE (2.5 mM) and Kraft lignin (0.25 mM) dissolved in 5% (1–2), 50% ethanol (3), and 5% DMSO (4) for 10 min.

In previous study, we showed that the artificial enzyme F43Y/T67R Mb exhibited peroxidase activity in aqueous buffer solution at ∼pH 5.5 toward oxidation of ABTS (Liu et al., 2019). Meanwhile, the solubility of lignin such as the model compounds GGE and Kraft lignin in aqueous solution is considerably low, which requires the addition of organic solvent such as ethanol and DMSO. However, the presence of organic solvent may cause side effects on the enzyme, such as the denaturation of enzyme and decrease of the enzymatic activity. To test the possibility, we evaluated the peroxidase activity of F43Y/T67R Mb in the oxidation of ABTS at pH 5.5, in the absence and presence of different amounts of ethanol or DMSO. Kinetic studies (Supplementary Figure 1) showed that the presence of 15% (v/v) ethanol has less effect (<5%) on the peroxidase activity (Figure 3A), with 20% (v/v) resulted in ∼15% loss of activity. Meanwhile, the presence of DMSO has more profound effect. For example, 5 and 20% (v/v) DMSO resulted in ∼40 and ∼90% loss of the activity, respectively (Figure 3B).

We also investigated the H_2_O_2_ dependence of the peroxidase activity of F43Y/T67R Mb in oxidation of ABTS (Figure 3C). The results showed that the enzyme exhibited a turnover number (*k*_*cat*_) of 39.2 ± 2.7 s^–1^, corresponding to a specific activity of ∼130.6 U/mg, which is close to that reported for the most efficient native enzyme, horseradish peroxidase (HRP) (*k*_*ca*__*t*_ = 52.5 ± 3.9 s^–1^) (Rodriguez-Lopez et al., 1996). Noted that no obvious inhibition effect was observed, even at a high concentration of 100 mM H_2_O_2_, suggesting the high tolerance of H_2_O_2_ for the enzyme. This property is distinct from that of other enzymes, such as an actinobacterial DyP-type peroxidase reported recently, with complete inhibition even at a low concentration of H_2_O_2_ (0.05 mM) (Musengi et al., 2020).

**FIGURE 4:**
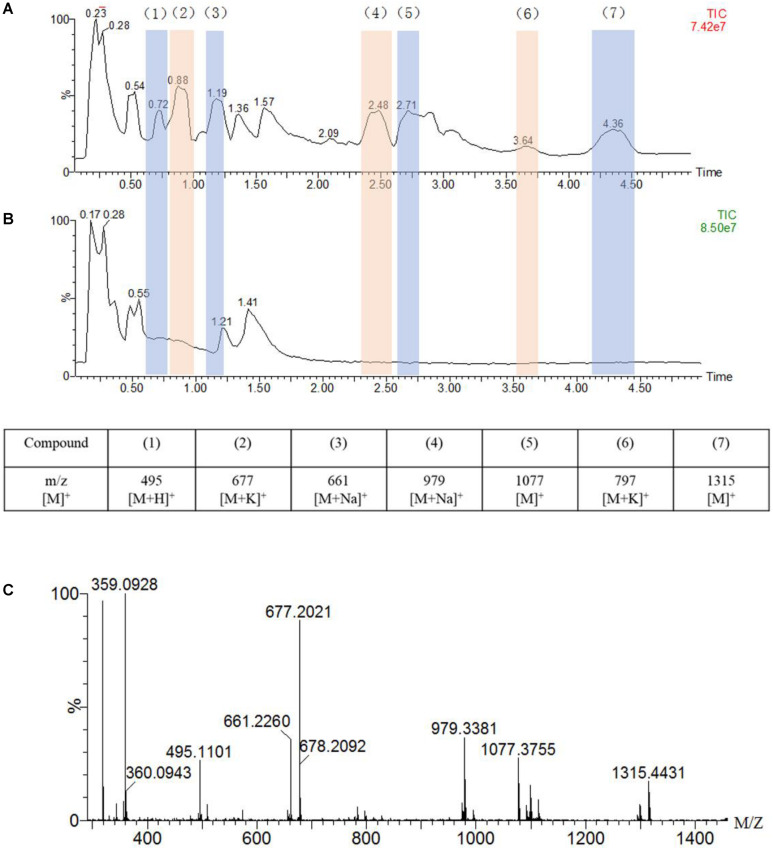
UPLC-MS traces of the oxidation of guaiacylglycerol-β-guaiacyl ether (GGE, 2.5 mM) catalyzed by F43Y/T67R Mb (5 μM) in the presence of H_2_O_2_ (2.0 mM) **(A)** or in the absence of H_2_O_2_ **(B)**, in potassium phosphate buffer (pH 5.5). Detected products are numbered and listed in the table below the chromatograms together with the corresponding m/z values. **(C)** Analysis of the oxidation products by ESI–MS spectrometry in a positive mode.

The lignin model compound GGE, with two monomers of coniferyl alcohol, was found to have a good solubility in aqueous solution containing ∼5% ethanol (Figures 1, 3D). However, Kraft lignin, due to large amounts of the three major monomers with a large average molecular mass of 10,000 Da, could not dissolve by addition of 50% ethanol (Figure 3). Instead, it has a good solubility by an addition of 5% DMSO (Figures 3D, 4), no need of higher concentrations such as 15% (Singh et al., 2013). Therefore, we chose to use the optimal reaction conditions for oxidation of GGE and Kraft lignin by the addition of 5% ethanol and DMSO, respectively, in following sections.

### Oxidation Products of GGE Catalyzed by F43Y/T67R Mb

The reaction of GGE by F43Y/T67R Mb allowed a more detailed study of the molecular site of action and catalytic mechanism. At first, we analyzed the titration of F43Y/T67R Mb solution with GGE in the absence or presence of H_2_O_2_ by using UV-Vis spectra. The results showed that, compared with the control in the absence of H_2_O_2_ (Supplementary Figure 2, black spectrum), the Soret peak of the substrate GGE at 276 nm and the Soret peak of the protein at 404 nm were reduced in reaction with H_2_O_2_ (Supplementary Figure 2, red spectrum). These observations suggest that the oxidation of GGE by F43Y/T67R Mb was happened. Therefore, we further studied the degradation products of GGE catalyzed by F43Y/T67R Mb using UPLC-ESI-MS.

As shown in Figure 4A, the UPLC-ESI-MS results revealed a broad product peaks in the range of m/z 400–1,400, which were higher than the molecular weight of the starting GGE [320 Da, observed, 359 Da, [M + K]^+^]. Not that the signal of GGE was observed at 0.55 min in the total ion chromatograph (TIC) spectrum (Figure 4B). Comparing to the control study in the absence of H_2_O_2_ (Figure 4B), the TIC spectrum of GGE degradation by F43Y/T67R Mb (Figure 4A) showed that seven major products (compounds 1 to 7) were formed, and these new signals emerged at retention time (RT) 0.72, 0.88, 1.19, 2.48, 2.71, 3.64, and 4.36 min, respectively, which were considered to be depolymerization and polymerization products. Moreover, ESI-MS analysis showed that the molecular weights of compounds 1 to 7 correspond to m/z 495, 677, 661, 979, 1,077, 797, and 1,315 Da, respectively (Figure 4C). Therefore, these result indicated that recombination of radical products was taken place, generating higher-molecular weight species. At the same time, the obtained UPLC-ESI-MS data could support each expected depolymerization and polymerization product formed by the C-C or C-O bond depolymerization of GGE by F43Y/T67R Mb, as discussed below.

The chemical structure of GGE is the major structural unit in Kraft lignin, and there are several possible sites for oxidation or oxidative cleavage (Yang et al., 2019). In this study, the detection of the product with a molecular weight of 495 m/z indicated that the F43Y/T67R Mb can indeed degrade GGE. At the same time, we observed the formation of macromolecular substances, which suggests that the polymer was produced by further coupling of the monomers catalyzed by the enzyme.

**FIGURE 5:**
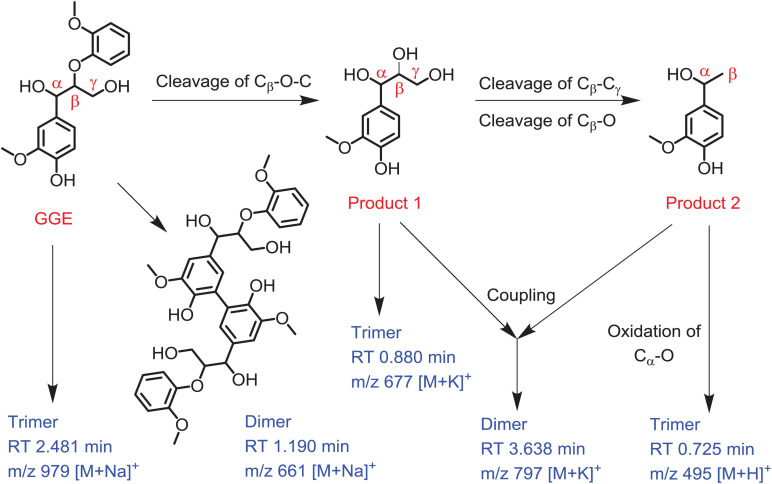
Depolymerization and polymerization of the guaiacylglycerol-β-guaiacyl ether (GGE) catalyzed by F43Y/T67R Mb. Product 1 is erythro-Guaiacylglycerol, and product 2 is 4-Hydroxy-3-methoxy-a-methylbenzyl alcohol. The corresponding mass of dimer and trimer are shown for clarification.

Based on the UPLC-ESI-MS data, we proposed the generation route of the reaction products. As shown in Figure 5, the substrate GGE may undergo dimerization and trimerization, producing dimer and trimer, which were detected at the RT 1.190 min and 2.481 min, respectively. Their corresponding molecular weights were matched molecular formula of C_34_H_38_O_12_ and C_51_H_56_O_18_, respectively. For oxidative cleavage, the monomers can hardly exist alone, and they will be recombined into new species. For example, by C_β_ -O-C cleavage of GGE, product 1 (erythro-Guaiacylglycerol) will be generated, which further forms a trimer, matching the expected molecular weight of C_30_H_38_O_15_, with a retention time of 0.880 min.

After the first cleavage of GGE, the reaction continued to release the product 2 (4-Hydroxy-3-methoxy-a-methylbenzyl alcohol) by cleavage of C_β_ -C_γ_ and C_β_ -O. Note that the products 1 and 2 may be coupled together and form a heterodimer, with a retention time of 3.638 min and an expected molecular weight of C_38_H_50_O_16_. Moreover, by further oxidation of C_α_ -O of product 2, the product may form a trimer, which has a RT of 0.725 min, with a molecular weight corresponding to the molecular formula of C_27_H_26_O_9_. From the proposed generation route of GGE, we can infer the possibility that the F43Y/T67R Mb could be used to attack Kraft lignin at the corresponding catalytic cracking site.

### Molecular Docking Structure of GGE-F43Y/T67R Mb Complex

**FIGURE 6:**
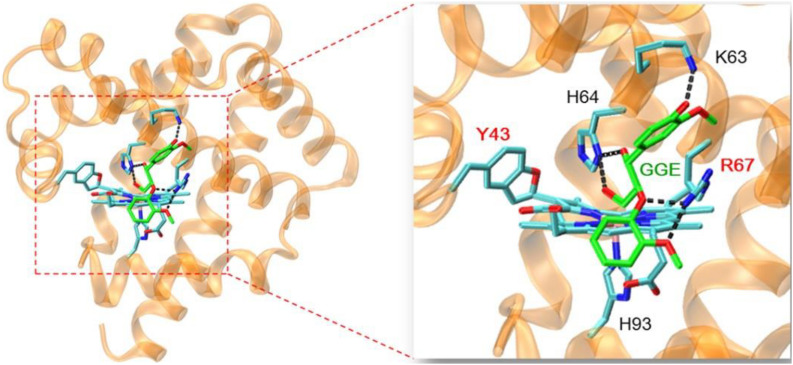
Molecular docking structure of GGE binding to F43Y/F46R Mb with the lowest binding energy. The structure of GGE is shown in green, and the H-bonding interactions between GGE and protein residues are indicated by dashed lines.

In order to provide structural information for GGE binding to F43Y/T67R Mb, the X-ray structure of ferric F43Y/T67R Mb was used as the initial structure for docking with the substrate. In the process of calculating the binding energy, we calculated ten sets of data (Supplementary Table 1), and selected the lowest energy to show the docking complex structure (Figure 6). The result showed that for the most stable structure, GGE binds to the protein surface of F43Y/F46R Mb close to heme active site, and interacts with the surface residues by hydrogen (H)-bonding interactions, including the side chains of Lys63, His64, and Arg67. These observations suggest that the binding model of GGE is favorable for bond cleavage by F43Y/T67R Mb, which also provides information for the binding site of Kraft lignin in enzymatic reactions.

### Activity Toward the Kraft Lignin Substrate

**FIGURE 7:**
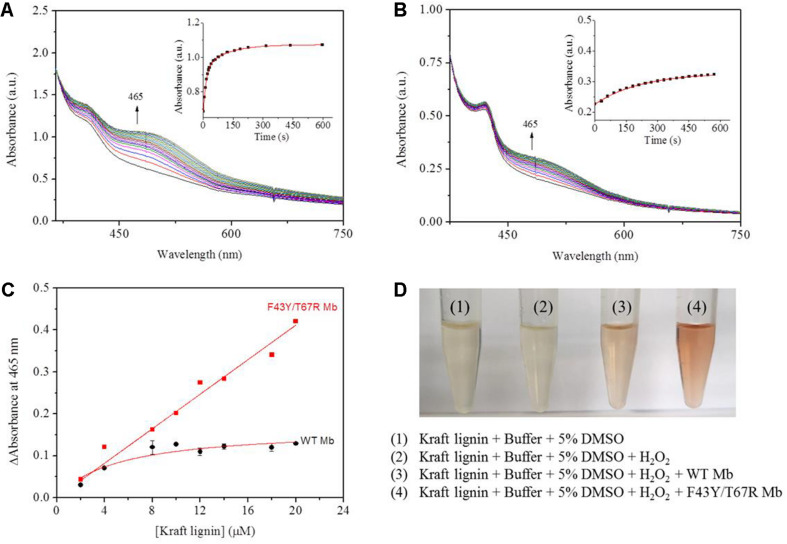
Kinetic studies of Kraft lignin (14 μM) in the presence of H_2_O_2_ (2.0 mM) catalyzed by 2 μM F43Y/T67R Mb **(A)** or WT Mb **(B)**. Time-dependent changes of the absorbance at 465 nm were shown as insets, and were fitted to the single-exponential decay function. **(C)** The changes of absorbance at 465 nm after 10 min with different concentrations of Kraft lignin, as catalyzed by F43Y/T67R Mb and WT Mb. The plots were fitted to the linear and Michaelis-Menten equations, respectively. **(D)** Visual appearance of Kraft lignin (14 μM) oxidation in the absence (1) and presence (2–4) of H_2_O_2_ (2.0 mM), WT Mb (3) and F43Y/T67R Mb (4) (2 μM) for 10 min.

According to the reaction conditions of GGE oxidation catalyzed by F43Y/T67R Mb, we performed the corresponding experiments for Kraft lignin. We first performed UV-Vis kinetic studies for Kraft lignin oxidation by monitoring the changes in the absorbance at 465 nm. As shown in Figure 7A, the oxidation rate catalyzed by F43Y/T67R Mb increased at first ∼60 s and reached a plateau after ∼300 s, with an obvious rate constant (*k*_*obs*_) of 0.070 ± 0.003 s^–1^. Control study using WT Mb showed that the reaction rate was considerably slow [*k*_*obs*_ = (4.5 ± 0.2) × 10^–3^ s^–1^, Figure 7B]. The concentration dependent of Kraft lignin was also investigated for both F43Y/T67R Mb and WT Mb. As shown in Figure 7C, after reaction for 10 min catalyzed by F43Y/T67R Mb, the absorbance at 465 nm increased with the increase of the concentration of Kraft lignin. Note that the activity (∼0.043 a.u./min) with 20 μM Kraft lignin was ∼1.7-fold higher than that reported recently for the wild-type peroxidase Dyp1B (0.0248 a.u./min) (Rahman Pour et al., 2019). Moreover, no inhibition effect was observed for F43Y/T67R Mb at substrate concentrations of Kraft lignin tested (2–20 μM). Meanwhile, in case of WT Mb under the same conditions, the increase of absorbance at 465 nm was much low, with an inhibition effect by Kraft lignin (>10 μM).

We also studied the visual appearances upon the oxidation of Kraft lignin (Figure 7D). After the assays, the resulting solutions appeared reddish in the treatment of Kraft lignin by WT Mb or F43Y/T67R Mb in presence of H_2_O_2_, with deeper color for the treatment by F43Y/T67R Mb, which was not observed for buffer solution containing Kraft lignin in the absence or present of H_2_O_2_. Therefore, these observations suggest that F43Y/T67R Mb in more effective in oxidation of Kraft lignin compared with the WT Mb.

To further investigate the involvement of F43Y/T67R Mb in Kraft lignin degradation, we analyzed the entire reaction products from the incubations of Kraft lignin with the enzyme in presence of H_2_O_2_ by mass spectrometry. As shown in Supplementary Figure 3A, the spectrum of the entire reaction revealed a large amount of low molecular weight products compared to the control reaction, i.e., the incubation of Kraft lignin with enzyme in the absence of H_2_O_2_ (Supplementary Figure 3B). Based on the ESI-MS analysis, we identified several degradation products with molecular weights of 300, 302, 330, 385, 390, and 434 Da, together with 495 Da, as observed for the oxidation of GGE (Figure 4C). According to the five subunits of Kraft lignin, the possible chemical structures of these low molecular weight products are shown in Table 1. Taken together, these results further verified the role of the F43Y/T67R Mb in generating low molecular weight lignin-derived compounds during the decomposition of Kraft lignin.

**TABLE 1:**
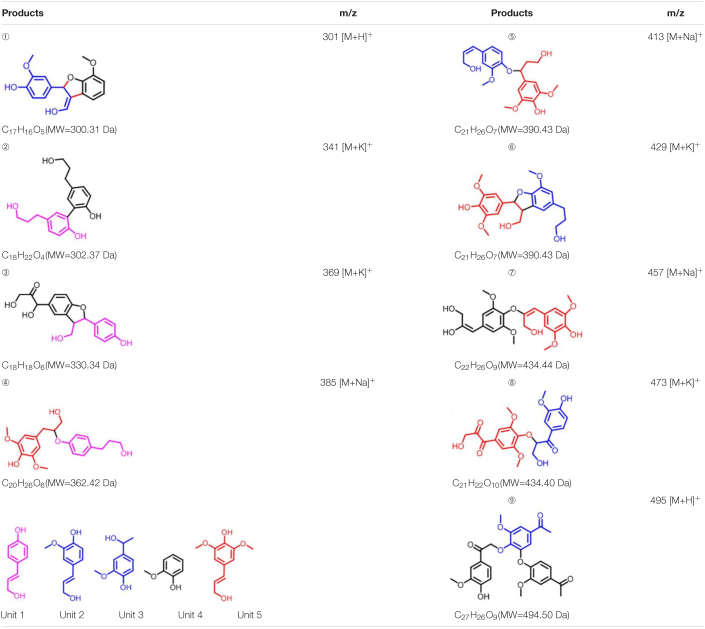
Estimated degradation products of Kraft lignin catalyzed by F43Y/T67R Mb based on the ESI-MS analysis.

*The five subunits of Kraft lignin are shown for clarification.*

## Conclusion

In this study, we successfully achieved the depolymerization of the model lignin dimer GGE and Kraft lignin, by using an artificial heme enzyme rationally designed in Mb, F43Y/T67R Mb. Kinetic UV-Vis studies and UPLC-ESI-MS analysis indicated that the artificial enzyme of F43Y/T67R Mb is effective in oxidation of both substrates. Based on the results, we proposed a plausible generation route of the reaction products catalyzed by the enzyme. Molecular modeling structure of the GGE-F43Y/T67R Mb complex provided further information for the binding site of the substrate, as well as bond cleavage in the enzymatic reactions. Moreover, we directly observed the color change of in the process of degradation of Kraft lignin catalyzed by F43Y/T67R Mb, with nine degradation products identified by the ESI-MS analysis. It should be noted that with a covalently linked heme group, the artificial enzyme of F43Y/T67R Mb exhibited considerable protein stability, with the tolerance of organic solvents such as 15% ethanol and 5% DMSO (v/v), and without the inhibition of the oxidant H_2_O_2_ even a high concentration of 100 mM. Since the artificial enzyme is readily to obtain by overexpression in *E. coli* cells, and the oxidant H_2_O_2_ is relatively cheap, this study is expected to provide an economic solution for the biodegradation of lignin, which is of great importance to the sustainable utilization of lignin.

## Data Availability Statement

The original contributions presented in the study are included in the article/Supplementary Material, further inquiries can be directed to the corresponding authors.

## Author Contributions

W-JG: data curation and writing – original draft. J-KX: data curation, supervision, and writing – review and editing. J-JLi: data curation. J-JLa: data curation and analysis. S-QG: data curation. G-BW: supervision. Y-WL: funding acquisition, supervision, and writing – review and editing. All authors contributed to the article and approved the submitted version.

## Conflict of Interest

The authors declare that the research was conducted in the absence of any commercial or financial relationships that could be construed as a potential conflict of interest.

## References

[B1] Ariyasu S. Stanfield J. K. Aiba Y. Shoji O. (2020). Expanding the applicability of cytochrome P450s and other haemoproteins. *Curr. Opin. Chem. Biol.* 59 155–163. 10.1016/j.cbpa.2020.06.010 32781431

[B2] Banerjee S. Mudliar S. Sen R. Giri B. Satpute D. Chakrabarti T. (2010). Commercializing lignocellulosic bioethanol: technology bottlenecks and possible remedies. *Biofuel. Bioprod. Bioref.* 4 77–93. 10.1002/bbb.188

[B3] Bugg T. D. H. Williamson J. J. Rashid G. M. M. (2020). Bacterial enzymes for lignin depolymerisation: new biocatalysts for generation of renewable chemicals from biomass. *Curr. Opin. Chem. Biol.* 55 26–33. 10.1016/j.cbpa.2019.11.007 31918394

[B4] Chan J. C. Paice M. Zhang X. (2020). Enzymatic oxidation of lignin: challenges and barriers toward practical applications. *ChemCatChem* 12 401–425. 10.1002/cctc.201901480

[B5] Chauhan P. S. (2020). Role of various bacterial enzymes in complete depolymerization of lignin: a review. *Biocatal. Agric. Biotechnol.* 23:101498. 10.1016/j.bcab.2020.101498

[B6] Chen C. Shrestha R. Jia K. Gao P. F. Geisbrecht B. V. Bossmann S. H. (2015). Characterization of Dye-decolorizing Peroxidase (DyP) from thermomonospora curvata reveals unique catalytic properties of A-type DyPs. *J. Biol. Chem.* 290 23447–23463. 10.1074/jbc.M115.658807 26205819 PMC4645587

[B7] Chen S.-F. Liu X.-C. Xu J.-K. Li L. Lang J.-J. Wen G.-B. (2021). Conversion of human neuroglobin into a multifunctional peroxidase by rational design. *Inorg. Chem.* 60 2839–2845. 10.1021/acs.inorgchem.0c03777 33539081

[B8] Davaritouchaee M. Hiscox W. C. Terrell E. Mancini R. J. Chen S. (2020). Mechanistic studies of milled and Kraft lignin oxidation by radical species. *Green Chem.* 22 1182–1197. 10.1039/C9GC04162A

[B9] Denisov I. G. Makris T. M. Sligar S. G. Schlichting I. (2005). Structure and chemistry of cytochrome P450. *Chem. Rev.* 105 2253–2277. 10.1021/cr0307143 15941214

[B10] Hrycay E. G. Bandiera S. M. (2012). The monooxygenase, peroxidase, and peroxygenase properties of cytochrome P450. *Arch. Biochem. Biophys.* 522 71–89. 10.1016/j.abb.2012.01.003 22266245

[B11] Humphrey W. Dalke A. Schulten K. (1996). VMD: visual molecular dynamics. *J. Mol. Graph.* 14 33–38. 8744570 10.1016/0263-7855(96)00018-5

[B12] Jiang Y. Wang C. Ma N. Chen J. Liu C. Wang F. (2020). Regioselective aromatic O-demethylation with an artificial P450BM3 peroxygenase system. *Catal. Sci. Technol.* 10 1219–1223. 10.1039/D0CY00241K

[B13] Kamimura N. Sakamoto S. Mitsuda N. Masai E. Kajita S. (2019). Advances in microbial lignin degradation and its applications. *Curr. Opin. Biotechnol.* 56 179–186. 10.1016/j.copbio.2018.11.011 30530243

[B14] Leonowicz A. Matuszewska A. Luterek J. Ziegenhagen D. Wojtas-Wasilewska M. Cho N. S. (1999). Biodegradation of lignin by white rot fungi. *Fungal Genet. Biol.* 27 175–185. 10.1006/fgbi.1999.1150 10441443

[B15] Li C. Zhao X. Wang A. Huber G. W. Zhang T. (2015). Catalytic transformation of lignin for the production of chemicals and fuels. *Chem. Rev.* 115 11559–11624. 10.1021/acs.chemrev.5b00155 26479313

[B16] Liao F. Xu J.-K. Luo J. Gao S.-Q. Wang X.-J. Lin Y.-W. (2020). Bioinspired design of an artificial peroxidase: introducing key residues of native peroxidases into F43Y myoglobin with a Tyr-heme cross-link. *Dalton Trans.* 49 5029–5033. 10.1039/d0dt00875c 32236202

[B17] Lin Y.-W. (2015). The broad diversity of heme-protein cross-links: an overview. *Biochim. Biophys. Acta* 1854 844–859. 10.1016/j.bbapap.2015.04.019 25916935

[B18] Lin Y.-W. (2018). Structure and function of heme proteins regulated by diverse post-translational modifications. *Arch. Biochem. Biophys.* 641 1–30. 10.1016/j.abb.2018.01.009 29407792

[B19] Lin Y.-W. (2020). Rational design of heme enzymes for biodegradation of pollutants toward a green future. *Biotechnol. Appl. Biochem.* 67 484–494. 10.1002/bab.1788 31175692

[B20] Lin Y.-W. (2021). Biodegradation of aromatic pollutants by metalloenzymes: a structural-functional-environmental perspective. *Coord. Chem. Rev.* 434:213774. 10.1016/j.ccr.2021.213774

[B21] Liu C. Xu J. Gao S.-Q. He B. Wei C.-W. Wang X.-J. (2018). Green and efficient biosynthesis of indigo from indole by engineered myoglobins. *RSC Adv.* 8 33325–33330. 10.1039/C8RA07825D PMC9086478 35548150

[B22] Liu C. Yuan H. Liao F. Wei C.-W. Du K.-J. Gao S.-Q. (2019). Unique Tyr-heme double cross-links in F43Y/T67R myoglobin: an artificial enzyme with a peroxidase activity comparable to that of native peroxidases. *Chem. Commun.* 55 6610–6613. 10.1039/C9CC02714A 31119219

[B23] Luo J. Du K.-J. Yuan H. Wei C.-W. Lang J.-J. Wen G.-B. (2020). Rational design of an artificial nuclease by engineering a hetero-dinuclear center of Mg-Heme in myoglobin. *ACS Catal.* 10 14359–14365. 10.1021/acscatal.0c04572

[B24] Mallinson S. J. B. Machovina M. M. Silveira R. L. Garcia-Borras M. Gallup N. Johnson C. W. (2018). A promiscuous cytochrome P450 aromatic O-demethylase for lignin bioconversion. *Nat. Commun.* 9:2487. 10.1038/s41467-018-04878-2 29950589 PMC6021390

[B25] Morris G. M. Huey R. Lindstrom W. Sanner M. F. Belew R. K. Goodsell D. S. (2009). AutoDock4 and AutoDockTools4: automated docking with selective receptor flexibility. *J. Comput. Chem.* 30 2785–2791. 19399780 10.1002/jcc.21256 PMC2760638

[B26] Musengi A. Durrell K. Prins A. Khan N. Agunbiade M. Kudanga T. (2020). Production and characterisation of a novel actinobacterial DyP-type peroxidase and its application in coupling of phenolic monomers. *Enzyme Microb. Technol.* 141:109654. 10.1016/j.enzmictec.2020.109654 33051013

[B27] Nastri F. Lista L. Ringhieri P. Vitale R. Faiella M. Andreozzi C. (2011). A heme-peptide metalloenzyme mimetic with natural peroxidase-like activity. *Chemistry* 17 4444–4453. 10.1002/chem.201003485 21416513

[B28] Paliwal R. Rawat A. P. Rawat M. Rai J. P. (2012). Bioligninolysis: recent updates for biotechnological solution. *Appl. Biochem. Biotechnol.* 167 1865–1889. 10.1007/s12010-012-9735-3 22639362

[B29] Picart P. de María P. D. Schallmey A. (2015). From gene to biorefinery: microbial β-etherases as promising biocatalysts for lignin valorization. *Front. Microbiol.* 6:916. 10.3389/fmicb.2015.00916 26388858 PMC4560021

[B30] Poulos T. L. (2014). Heme enzyme structure and function. *Chem. Rev.* 114 3919–3962. 10.1021/cr400415k 24400737 PMC3981943

[B31] Rahman Pour R. Ehibhatiomhan A. Huang Y. Ashley B. Rashid G. M. Mendel-Williams S. (2019). Protein engineering of *Pseudomonas* fluorescens peroxidase Dyp1B for oxidation of phenolic and polymeric lignin substrates. *Enzyme Microb. Technol.* 123 21–29. 10.1016/j.enzmictec.2019.01.002 30686347

[B32] Rahmanpour R. Rea D. Jamshidi S. Fulop V. Bugg T. D. H. (2016). Structure of Thermobifida fusca DyP-type peroxidase and activity towards Kraft lignin and lignin model compounds. *Arch. Biochem. Biophys.* 594 54–60. 26901432 10.1016/j.abb.2016.02.019

[B33] Rodriguez-Lopez J. N. Smith A. T. Thorneley R. N. (1996). Role of arginine 38 in horseradish peroxidase. A critical residue for substrate binding and catalysis. *J. Biol. Chem.* 271 4023–4030. 8626735 10.1074/jbc.271.8.4023

[B34] Schuttelkopf A. W. van Aalten D. M. F. (2004). PRODRG: a tool for high-throughput crystallography of protein-ligand complexes. *Acta Crystallogr. D Biol. Crystallogr.* 60 1355–1363. 10.1107/S0907444904011679 15272157

[B35] Singh R. Grigg J. C. Qin W. Kadla J. F. Murphy M. E. Eltis L. D. (2013). Improved manganese-oxidizing activity of DypB, a peroxidase from a lignolytic bacterium. *ACS Chem. Biol.* 8 700–706. 10.1021/cb300608x 23305326 PMC3631457

[B36] Stergiou D. V. Veltsistas P. G. Prodromidis M. I. (2008). An electrochemical study of lignin films degradation: proof-of-concept for an impedimetric ozone sensor. *Sens. Actuators B Chem.* 129 903–908. 10.1016/j.snb.2007.10.001

[B37] Upton B. M. Kasko A. M. (2016). Strategies for the conversion of lignin to high-value polymeric materials: review and perspective. *Chem. Rev.* 116 2275–2306. 10.1021/acs.chemrev.5b00345 26654678

[B38] Urayama P. Phillips G. N. Jr. Gruner S. M. (2002). Probing substates in sperm whale myoglobin using high-pressure crystallography. *Structure* 10 51–60. 10.1016/S0969-2126(01)00699-2 11796110

[B39] Wang Y. Lan D. Durrani R. Hollmann F. (2017). Peroxygenases en route to becoming dream catalysts. What are the opportunities and challenges? *Curr. Opin. Chem. Biol.* 37 1–9. 10.1016/j.cbpa.2016.10.007 27992798

[B40] Xu J. Wang C. Cong Z. (2019). Strategies for substrate-regulated P450 catalysis: from substrate engineering to Co-catalysis. *Chemistry* 25 6853–6863. 10.1002/chem.201806383 30698852

[B41] Yan D.-J. Li W. Xiang Y. Wen G.-B. Lin Y.-W. Tan X. (2015). A novel tyrosine-heme C-O covalent linkage in F43Y myoglobin: a new post-translational modification of heme proteins. *Chembiochem* 16 47–50. 10.1002/cbic.201402504 25392956

[B42] Yang J. Gao T. Zhang Y. Wang S. Li H. Li S. (2019). Degradation of the phenolic beta-ether lignin model dimer and dyes by dye-decolorizing peroxidase from Bacillus amyloliquefaciens. *Biotechnol. Lett.* 41 1015–1021. 10.1007/s10529-019-02696-0 31134460

[B43] Yin L.-L. Yuan H. Liu C. He B. Gao S.-Q. Wen G.-B. (2018). A rationally designed myoglobin exhibits a catalytic dehalogenation efficiency more than 1000-Fold that of a native dehaloperoxidase. *ACS Catal.* 8 9619–9624. 10.1021/acscatal.8b02979

[B44] Yoshikawa T. Yagi T. Shinohara S. Fukunaga T. Nakasaka Y. Tago T. (2013). Production of phenols from lignin via depolymerization and catalytic cracking. *Fuel Process. Technol.* 108 69–75. 10.1016/j.fuproc.2012.05.003

[B45] Zakzeski J. Bruijnincx P. C. Jongerius A. L. Weckhuysen B. M. (2010a). The catalytic valorization of lignin for the production of renewable chemicals. *Chem. Rev.* 110 3552–3599. 10.1021/cr900354u 20218547

[B46] Zakzeski J. Jongerius A. L. Weckhuysen B. M. (2010b). Transition metal catalyzed oxidation of Alcell lignin, soda lignin, and lignin model compounds in ionic liquids. *Green Chem.* 12 1225–1236. 10.1039/C001389G

[B47] Zhang C. Wang F. (2020). Catalytic lignin depolymerization to aromatic chemicals. *Acc. Chem. Res.* 53 470–484. 10.1021/acs.accounts.9b00573 31999099

[B48] Zhang P. Xu J. Wang X.-J. He B. Gao S.-Q. Lin Y.-W. (2019). The third generation of artificial dye-decolorizing peroxidase rationally designed in myoglobin. *ACS Catal.* 9 7888–7893. 10.1021/acscatal.9b02226

[B49] Zhang P. Yuan H. Xu J. Wang X.-J. Gao S.-Q. Tan X. (2020). A catalytic binding site together with a distal tyr in myoglobin affords catalytic efficiencies similar to natural peroxidases. *ACS Catal.* 10 891–896. 10.1021/acscatal.9b05080

[B50] Zhang X. Tu M. Paice M. G. (2011). Routes to potential bioproducts from lignocellulosic biomass lignin and hemicelluloses. *BioEnergy Res.* 4 246–257. 10.1007/s12155-011-9147-1

