# Supplementary material for: Biotransformation of Lignin by an Artificial Heme Enzyme Designed in Myoglobin With a Covalently Linked Heme Group
Source: Front Bioeng Biotechnol. 2021 May 31;9:664388. doi: 10.3389/fbioe.2021.664388 (PMC8201792; doi:10.3389/fbioe.2021.664388)
Supplement: Supplementary file 1 [file Data_Sheet_1.PDF]

Supporting Information for

Biotransformation of lignin by an artificial heme enzyme designed in myoglobin with a covalently linked heme group

Wen-Jie Guo,[a] Jia-Kun Xu,[b*] Jing-Jing Liu,[a] Jia-Jia Lang,[c] Shu-Qin Gao,[c] Ge-Bo Wen,[c] and Ying-Wu Lin [a,c*]

[a] School of Chemistry and Chemical Engineering, University of South China, Hengyang 421001, China

[b] Key Lab of Sustainable Development of Polar Fisheries, Ministry of Agriculture and Rural Affairs, Yellow Sea Fisheries Research Institute, Chinese Academy of Fishery Sciences, Lab for Marine Drugs and Byproducts of Pilot National Lab for Marine Science and Technology, Qingdao 266071, China

[c] Laboratory of Protein Structure and Function, University of South China Medical School, Hengyang 421001, China

Corresponding authors:

*E-mail: ywlin@usc.edu.cn (Y.-W. Lin); xujk@ysfri.ac.cn (J.-K. Xu)

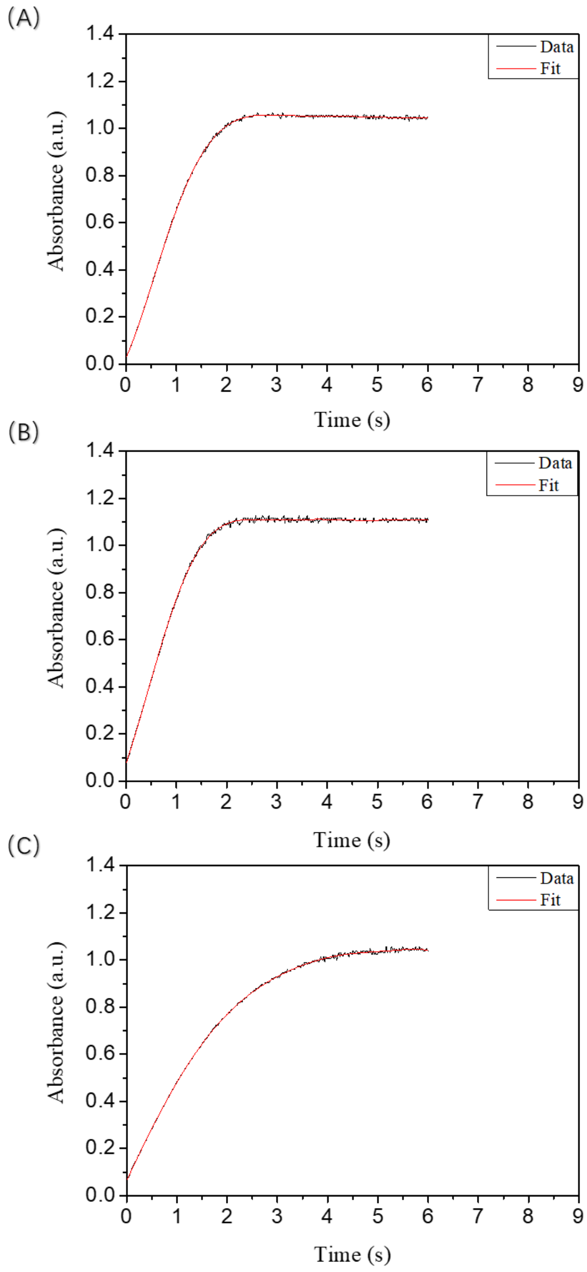

Fig. S1 Steady state kinetic data for the oxidation of ABTS (0.1 mM) catalyzed by F43Y/T67R Mb (2 μM) in the absence (A), and presence of ethanol (5%, v/v, B) and DMSO (5%, v/v, C), which were monitored by the absorbance changes at 660 nm. The spectra (black) were fitted to a single exponential with good fit (red). The obtained rate constants were k_{obs} = 1.14 s^{-1}, k_{obs} = 1.28 s^{-1}, k_{obs} = 0.59 s^{-1}, respectively.

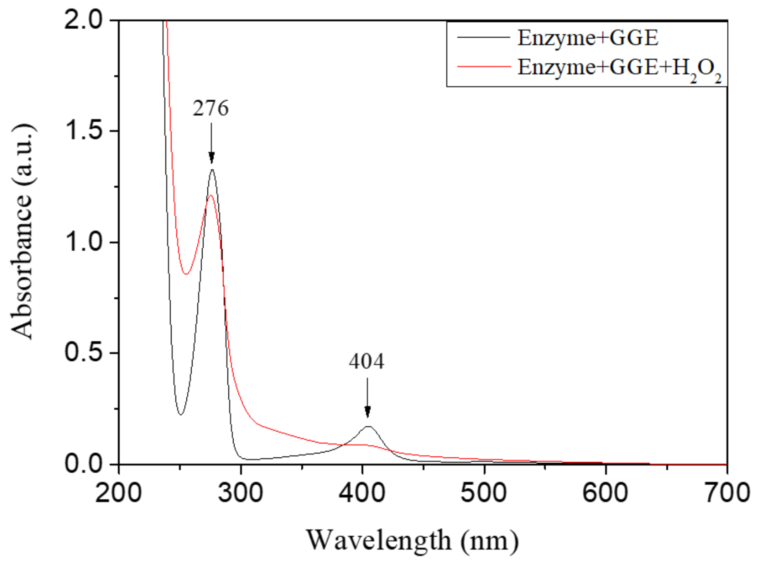

Fig. S2 UV-Vis spectra of F43Y/T67R Mb (0.5 μM) titrated with GGE (1 mM) in the absence (black spectrum) or presence of H_2O_2 (1 mM, red spectrum).

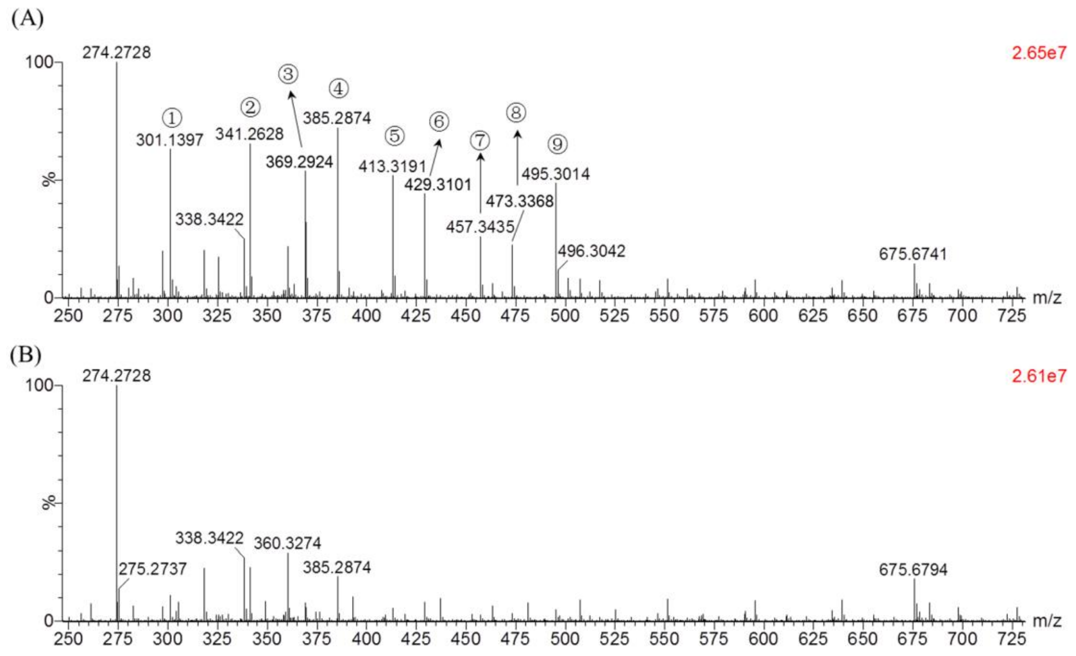

Fig. S3 Mass spectra of the oxidation products of Kraft lignin (25 μM) in the presence (A) and absence (B) of F43Y/T67R Mb (2 μM) with H_2O_2 (2 mM) as an oxidant.

Table S1. Docking results of GGE binding to F43Y/T67R Mb.

Model	$E_{binding}$ [a]	$E_{inter\text{-}mol}$ [b]	E_{vdw} [c]	E_{elec} [d]
1	-4.91	-7.89	-4.86	-0.46
2	-4.88	-7.86	-3.95	-0.83
3	-4.84	-7.83	-3.74	-0.74
4	-4.51	-7.49	-2.57	-0.38
5	-4.45	-7.43	-3.76	-0.86
6	-4.45	-7.44	-5.23	-0.81
7	-4.42	-7.41	-4.46	-0.56
8	-4.35	-7.33	-3.38	-0.63
9	-4.34	-7.33	-3.11	-0.41
10	-4.33	-7.32	-4.59	-0.75

[a] Binding energy. [b] Intermolecular energy. [c] van der Waals energies. [d] Electrostatic interactions.
